# The attachment process and physiological properties of *Escherichia coli* O157:H7 on quartz

**DOI:** 10.1186/s12866-020-02043-8

**Published:** 2020-11-19

**Authors:** Liliang Wang, Yichao Wu, Peng Cai, Qiaoyun Huang

**Affiliations:** State Key Laboratory of Agricultural Microbiology, College of Resources and Environment, Huazhong Agricultural University, Wuhan, 430070 China

**Keywords:** *Escherichia coli* O157:H7, Chemotaxis, Rcs system, Fermentative route, Stress susceptibility

## Abstract

**Background:**

Manure application and sewage irrigation release many intestinal pathogens into the soil. After being introduced into the soil matrix, pathogens are commonly found to attach to soil minerals. Although the survival of mineral-associated *Escherichia coli* O157:H7 has been studied, a comprehensive understanding of the attachment process and physiological properties after attachment is still lacking.

**Results:**

In this study, planktonic and attached *Escherichia coli* O157:H7 cells on quartz were investigated using RNA sequencing (RNA-seq) and the isobaric tagging for relative and absolute quantitation (iTRAQ) proteomic method. Based on the transcriptomic and proteomic analyses and gene knockouts, functional two-component system pathways were required for efficient attachment; chemotaxis and the Rcs system were identified to play determinant roles in *E. coli* O157:H7 attachment on quartz. After attachment, the pyruvate catabolic pathway shifted from the tricarboxylic acid (TCA) cycle toward the fermentative route. The survival rate of attached *E. coli* O157:H7 increased more than 10-fold under penicillin and vancomycin stress and doubled under alkaline pH and ferric iron stress.

**Conclusions:**

These results contribute to the understanding of the roles of chemotaxis and the Rcs system in the attachment process of pathogens and indicate that the attachment of pathogens to minerals significantly elevates their resistance to antibiotics and environmental stress, which may pose a potential threat to public health.

**Supplementary Information:**

The online version contains supplementary material available at 10.1186/s12866-020-02043-8.

## Background

*Escherichia coli* O157:H7 is one of the most ubiquitous foodborne zoonotic pathogens [1–3]. Sewage irrigation and manure application to soil have caused the pathogen to seep into soil matrices [4]. After introduction into the soil environment, this pathogen can be further transmitted to sediment, water, crops, and livestock, which have been directly linked to many cases of *E. coli* O157:H7 infection [5, 6]. Bacteria in the soil matrix proliferate in both sessile and planktonic states. In the environment, the sessile state is the dominant lifestyle of microorganisms, providing physical, chemical, and biological protection to the microorganisms [7–9]. Therefore, understanding the attachment process and physiological features of surface-attached *E. coli* O157:H7 in the soil environment is key to assessing the potential risks of this bacterium to public health [10].

As a general observation, most regulatory pathways participating in attachment are part of two-component signal transduction systems (TCS), which consist of a membrane-anchored signal sensor and a cytoplasmic response regulator [11, 12]. For example, the response regulator gene *rcsB* promotes the attachment of *E. coli* O157:H7 by downregulating flagellum-related genes but upregulating genes linked to the stress response [13]. Additionally, a quorum sensing (QS) system has been shown to promote *E. coli* O157:H7 attachment in the sand column by increasing the autoinducer-2 (AI-2) and exopolysaccharide content [14, 15]. In *E. coli* MG1655, overexpression of *ompR* (part of the EnvZ-OmpR system) increases initial attachment in sand columns because the OmpR protein promotes the formation of curli and extracellular structures involved in bacterial attachment [16]. Furthermore, the most intensively studied TCS, CpxAR, promotes attachment by inhibiting the expression of curli and flagella in *E. coli* [11]. A *cpxR* mutant exhibited a decreased level of attachment on quartz than wild-type *E. coli* K-12 [17]. These studies have verified the contribution of specific TCSs to *E. coli* attachment. However, the complete process by which *E. coli* O157:H7 integrates different TCSs during attachment to soil minerals is still poorly understood.

The physiological activity of surface-attached *E. coli* O157:H7 in the soil has changed significantly, and one of the main factors influencing the physiological state is the soil mineralogical properties [18, 19]. Fecal *E. coli* was found to preferentially attach to soil particles with a size range of 16–30 μm [20]. The survival period of surface-attached *E. coli* O157:H7 is greater than that of planktonic cells, which is attributed to elevated ATP levels and altered metabolic activities [21]. Additionally, it has been demonstrated that surface attachment promotes biofilm formation by enhancing colanic acid biosynthesis [22]. In addition, surface-attached *E. coli* O157:H7 improves the detoxification effect dependent on NADPH and the response to acidic pH and membrane stress [23]. While the survival of surface-associated *E. coli* O157:H7 has been studied, a comprehensive understanding of bacterial physiological properties after attachment to soil minerals is lacking.

Thus, the objective of this study was to investigate the attachment process and physiological features of *E. coli* O157:H7 during attachment to soil minerals. Quartz is a common inert mineral in soil, and the attachment of *E. coli* on quartz depends on the characteristics of the cells rather than the size of the particles [24]. Therefore, choosing quartz as the attachment surface is helpful for separation of the attached cells and analysis of the influence of cell properties on the attachment process. In this study, RNA-seq transcriptomics combined with an iTRAQ proteomic approach was performed to analyze differentially expressed genes and proteins in planktonic and attached *E. coli* O157:H7 and to provide new insights into the attachment process. The regulatory system for induction of irreversible surface attachment was further explored. The antibiotic susceptibility and resistance to environmental stress of surface-associated bacteria were characterized. The results provide new insights into the mechanism of bacterial attachment on mineral surfaces and the corresponding physiological responses. We hypothesized that the TCS of *E. coli* O157:H7 may dominate the attachment process, and the metabolic activity of the bacteria would decrease after attachment.

## Results

### Physiological differences at the transcriptome and proteome levels

To characterize the physiological changes of quartz-associated *E. coli* O157:H7, combined transcriptome and proteome analyses were carried out. A total of 554 out of 4814 genes (11.5% of the total genes screened) were significantly differentially transcribed. Approximately 6.7% (323 of 4814) of the genes were upregulated in attached cells, whereas 4.8% (231 of 4814) of the genes were downregulated by more than 2-fold (Figure S1), compared to the levels in planktonic *E. coli* O157:H7.

**Fig. 1:**
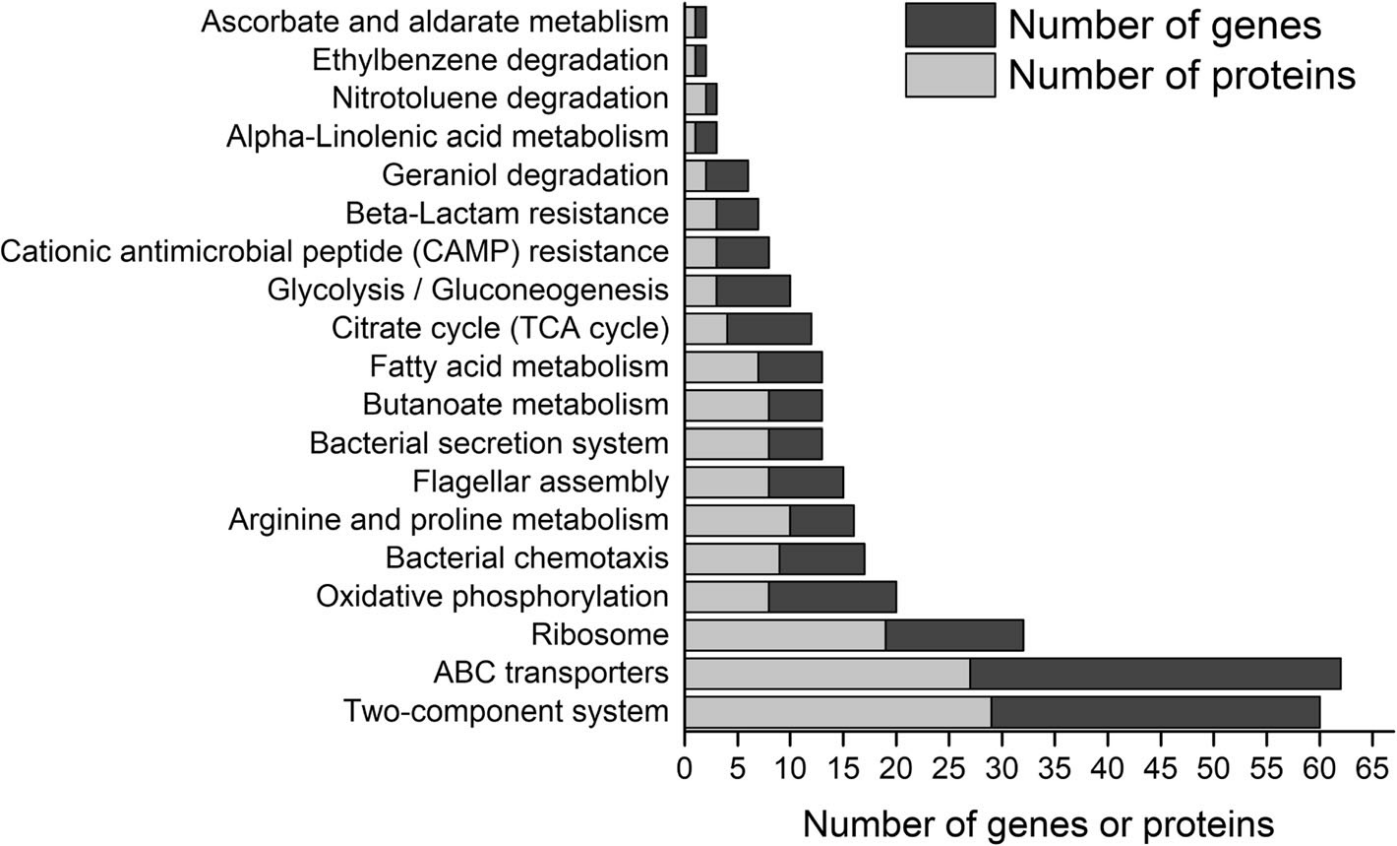
The numbers of genes and proteins that were differentially expressed during attachment mainly assigned to the 19 pathways. Genes and proteins were categorized according to their regulatory pathways from the KEGG pathway database for *E. coli* O157:H7

iTRAQ-based proteomic analysis identified 18,157 unique peptides associated with 2782 proteins (Table S1). Among the identified proteins, 91 were upregulated and 260 were downregulated by more than 1.5-fold (Figure S1). As shown in Fig. 1, according to the KEGG classification, the genes and proteins differentially expressed during attachment mainly belonged to ABC transporters (27 proteins, 35 genes), two-component system (29 proteins, 31 genes), ribosome (19 proteins, 13 genes), oxidative phosphorylation (8 proteins, 12 genes), chemotaxis system (9 proteins, 8 genes), arginine and proline metabolism (10 proteins, 6 genes), flagella assembly (8 proteins, 7 genes), bacterial secretion system (8 proteins, 5 genes), methyl butyrate metabolism (8 proteins, 5 genes), fatty acid metabolism (7 proteins, 6 genes), tricarboxylic acid cycle (4 proteins, 8 genes), glycolysis/gluconeogenesis (3 proteins, 7 genes), cationic antimicrobial peptide resistance (3 proteins, 5 genes) and β-lactam antibiotic resistance (3 proteins, 4 genes).

### Role of two-component signal transduction systems (TCSs) in bacterial attachment to quartz

**Fig. 2:**
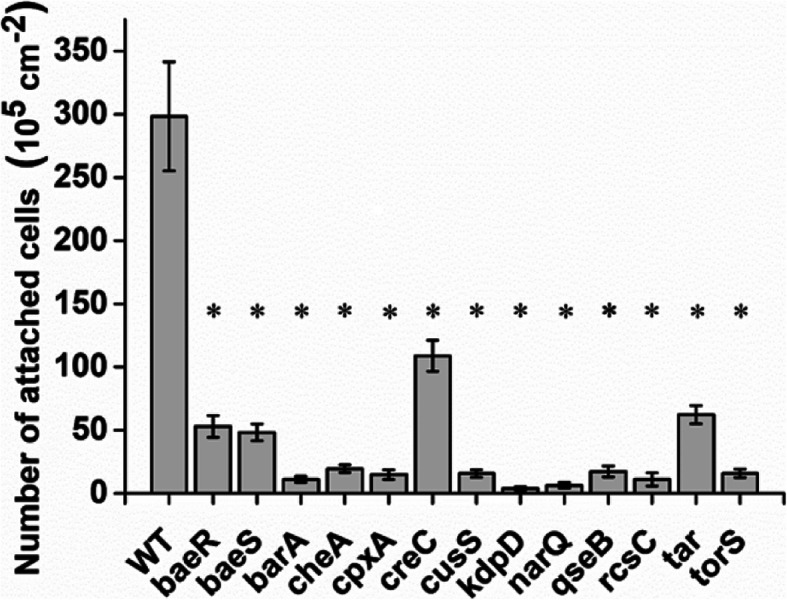
Attachment of wild-type and mutant *E. coli* O157:H7 strains to quartz. The numbers of attached cells were determined by QCM-D. The asterisk represents a significant difference between the wild-type and mutant strains (*p* < 0.05)

Twenty-nine proteins and thirty-one genes that were significantly differentially regulated in quartz-attached cells belonged to TCSs (Fig. 1), suggesting an important role in attachment. Gene knockout mutants of the selected TCS genes were constructed to test the importance of TCS on the attachment of *E. coli* O157:H7 on quartz (Table S2). We constructed 13 knockout mutants of TCS genes, including *baeR* and *baeS*, which regulate the multidrug efflux system; *barA*, which regulates carbon storage; *cheA* and the sensor *tar*, which regulate chemotaxis; *cpxA*, which regulates surface protein folding; the global carbon metabolism regulator *creC*; *cusS*, which responds to copper and silver ions; *kdpD*, which responds to K^+^; *narQ*, which regulates nitrogen metabolism; *torS*, a trimethylamine N-oxide metabolism-regulating gene; *qseB*, which regulates quorum sensing; and *rcsC*, which regulates capsular polysaccharide secretion. Q-CMD was used to explore the attachment capacity of each mutant or wild-type on the quartz surface. The results are shown in Fig. 2. The attachment capacity of the chemotaxis (*cheA*) and Rcs (*rcsC*) mutant strains was reduced by 93 and 96%, respectively; the attachment capacity of the *creC* mutant strain was reduced by 64%, the attachment capacity of the *baeS*, *baeR* and *tar* mutant strains was reduced by 79–84%, and the attachment capacity of the remaining mutant strains was reduced by more than 94%. These results prove that functional TCSs were required for efficient attachment.

### Differences in the expression of genes and proteins related to the attachment process of *E. coli* O157:H7

**Fig. 3:**
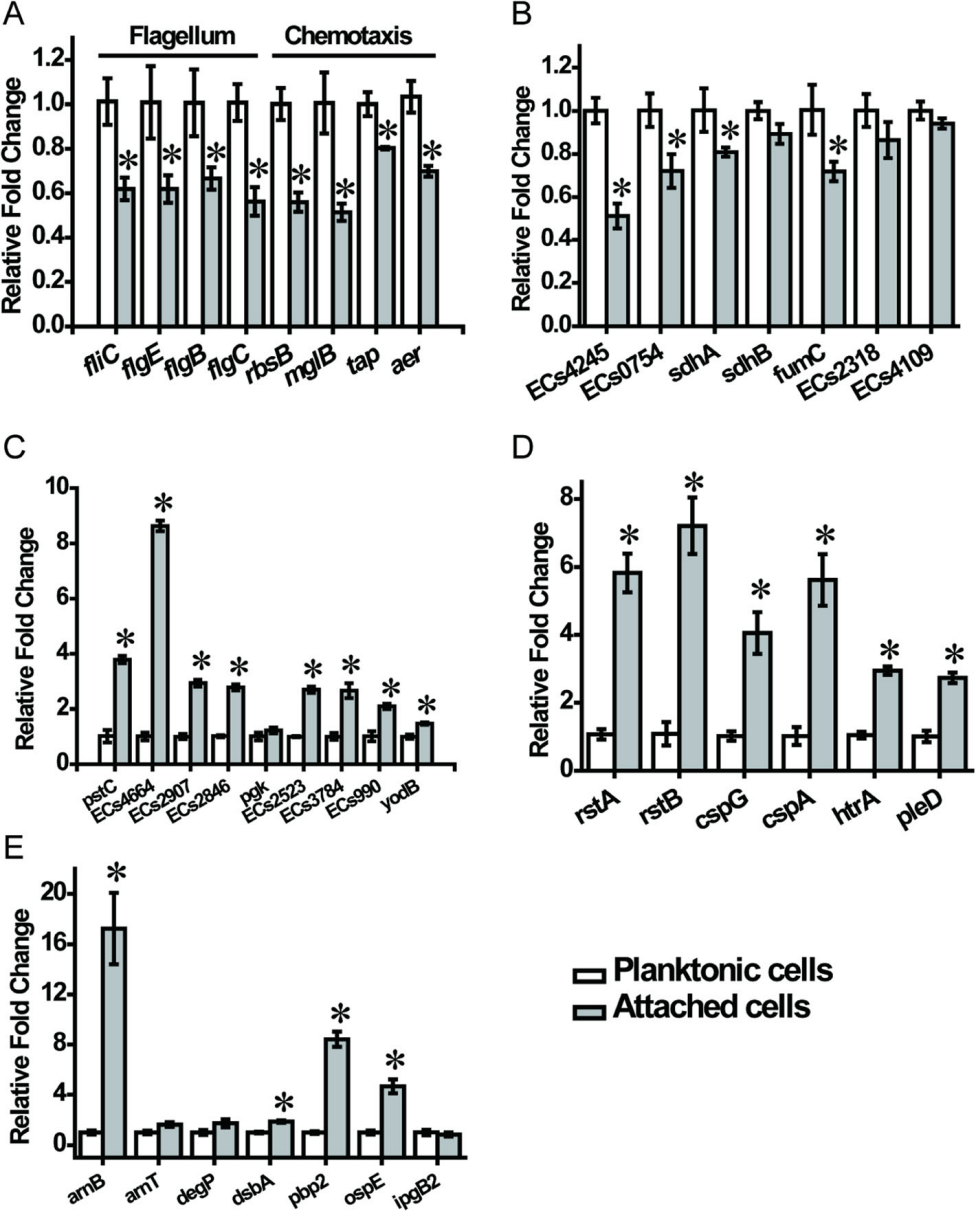
qPCR analysis of selected gene expression in quartz-attached and planktonic *E. coli* O157:H7. The attached cells were compared with the planktonic cells. Data were normalized to *gapA* expression levels. These genes were grouped by the following functional pathways: flagellar motility and bacterial chemotaxis (**a**), TCA cycle (**b**), fermentation metabolism (**c**), general stress response (**d**), and antibiotic resistance and pathogenicity (**e**). The asterisk represents a significant difference between the wild-type and mutant strains (*p* < 0.05)

Table S3 shows that attachment significantly inhibited the chemotaxis of *E. coli* O157:H7, and the expression of the sensor proteins Tsr (− 0.5), Tar (− 0.6), DppA (− 0.6), Tap (− 0.7), and Aer (− 0.9) and the regulatory proteins CheA (− 0.8), CheB (− 0.5), and CheW (− 0.4) decreased significantly. As shown in Table 1, after attachment, the genes *ECs0893* (3.2), *Ecs4884* (1.2), *Ecs4425* (2.0), *Ecs5326* (4.8), *Ecs2314* (2.0), and *Ecs2315* (1.4) and the protein KdsC (0.7) related to lipopolysaccharide (LPS) biosynthesis were upregulated, and the expression of the genes *Ecs5326* (4.8), *Ecs2314* (2.0) and *Ecs2315* (1.4) related to the Rcs system increased. Additionally, the structural proteins FlhD (− 0.8), FliM (− 0.2), FliF (− 0.4), FlgE (− 0.8), and FliC (− 1.2) and the genes *Ecs1451* (− 3.9), *Ecs1452* (− 2.7) and *Ecs1454* (− 2.4) related to flagella were downregulated in attached cells. The differential transcription of chemotaxis- and flagellum-related genes was confirmed by RT-qPCR analysis (Fig. 3a).

**Table 1:** Differentially altered proteins and genes associated with attachment

	Attached/Planktonic		Gene or protein description
	Protein	Gene ID	
Lipopolysaccharide biosynthesis	KdsC (0.7)		3-deoxy-D-manno-octulosonate 8-phosphate phosphatase
		*ECs0893* (3.2)	Phosphoethanolamine transferase
		*ECs4884* (1.2)	Membrane protein
		*ECs4425* (2.0)	Phosphoethanolamine transferase
Rcs system		*ECs5326* (4.8)	BglJ family transcriptional regulator RcsB
		*ECs2314* (2.0)	DNA-binding transcriptional regulator RstA
		*ECs2315* (1.4)	Sensor protein RstB
Flagellar assembly	FlhD (− 0.8)		Flagellar transcriptional regulator FlhD
	FliM (−0.2)		Flagellar motor switch protein FliM
	FliF (−0.4)		Flagellar M-ring protein FliF
		*ECs1451* (−3.9)	Flagellar basal-body rod protein FlgB
		*ECs1452* (−2.7)	Flagellar basal body rod protein FlgC
	FlgE (−0.8)	*ECs1454* (−2.4)	Flagellar hook protein FlgE
	FliC (−1.2)		Flagellin FliC

### Differences in the expression of genes and proteins related to metabolism

Gene set enrichment analysis (GSEA) was performed using the GO and KEGG databases. Table S4 shows that two GO terms were significantly different in both transcriptomic and proteomic data. Fatty acid beta-oxidation was significantly suppressed in both transcriptomic and proteomic data during the attachment phase. After attachment, the proteins FadM (− 0.4), FadB (− 0.6), FadE (− 0.7), and FadA (− 0.6) and genes *Ecs4774* (− 2.5), *Ecs0248* (− 1.9), *Ecs4773* (− 2.9), *Ecs3225* (− 1.5), *Ecs3224* (− 1.9), and *Ecs2514* (− 1.8) involved in fatty acid β-oxidation were significantly downregulated, indicating that fatty acid β-oxidation activity decreased. In addition, during attachment, the proteins AstB (− 0.6), AstA (− 0.1), PuuA (− 1.0), PuuB (− 1.3), PuuC (− 0.9), SpeG (− 0.6), PatA (− 0.8), SpeE (− 0.3), LysA (− 0.6) and XthA (− 0.5) involved in arginine and proline metabolism were significantly downregulated, indicating that the metabolic activity of arginine and proline was reduced (Table S3). Table S5 also shows that the transcription level of genes encoding tricarboxylic acid (TCA) cycle-related enzymes, including phosphoenolpyruvate carboxykinase (*Ecs4245* (− 2.3)), succinyl-CoA synthase α subunit (*Ecs0754* (− 1.0)), succinate dehydrogenase/fumarate reductase (*Ecs0748* (− 1.1) and *Ecs0749* (− 1.1)), fumarate hydrolase (*Ecs2317* (− 1.8), *Ecs2318* (− 1.7) and *Ecs5132* (− 2.4)), malate dehydrogenase (*Ecs4109* (− 1.0)) and the epsilon subunit of ATP synthase (*Ecs4673* (− 2.3)), is reduced after attachment, and the fumarate reductase FrdD (− 0.9) and malate dehydrogenase Mdh (− 0.7) are also downregulated, indicating that TCA cycle activity is reduced after attachment. However, the expression of the phosphoglycerate kinase Pgk (1.3) and glucokinase (1.3) was upregulated, indicating that glycolysis/glycogenesis was enhanced in attached cells. In addition, the genes *Ecs2523* (1.4), *Ecs3784* (1.9) and *Ecs0990* (1.3) involved in the biosynthesis and degradation of L-serine were upregulated, indicating that the metabolic activity of L-serine was enhanced in attached cells. We confirmed through RT-qPCR experiments that attachment inhibited the expression of the TCA-related genes *Ecs4245*, *Ecs0754*, s*dhA*, *sdhB*, *fumC*, *Ecs2318*, and *Ecs4109* and promoted the expression of the fermentation metabolism-related genes *pstC*, *Ecs4664*, *Ecs2907*, *Ecs2846*, *pgk*, *Ecs2523*, *Ecs3784*, *Ecs0990*, and *yodB* (Fig. 3b and c).

### Tolerance of environmental stress after *E. coli* O157:H7 attachment

**Fig. 4:**
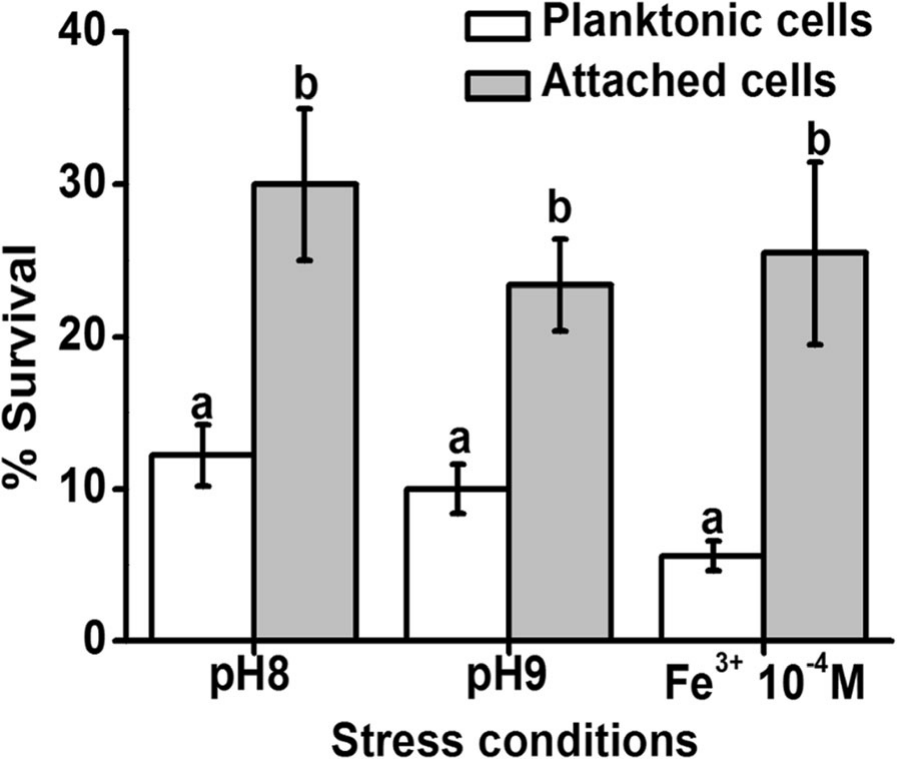
Susceptibility of planktonic and attached cells to alkaline pH and Fe^3+^ after 1 h of attachment. The planktonic and attached cells were collected after 1 h of attachment and were added to physiological saline with the pH adjusted to 8 and 9 by adding NaOH and 1 × 10^− 4^ M ferric iron for another hour. The number of viable cells was measured by dilution plating. The y-axis in the graph represents the proportion of viable cells after the addition of stimuli to the initial sample. Different letters indicate significant differences

As shown in Table S6, the expression levels of the genes *Ecs2315* (1.4) and *Ecs2314* (2.0) encoding the histidine kinase RstB and DNA-binding transcription regulator RstA were upregulated in attached cells, and the gene *Ecs0165* (1.8) related to the folding and degradation of cell envelope proteins was upregulated. These genes are related to the general stress response of the bacterium, indicating that attachment enhances the resistance of *E. coli* O157:H7 to general environmental stress. In addition, the expression of the gene *Ecs0623* (2.0) encoding the ferric iron-binding protein PfeA is upregulated. Table S4 also shows that the expression of the genes *Ecs4189* (2.4), *Ecs1480* (3.2) and *Ecs0154* (1.2) related to ferric iron metabolism is upregulated, suggesting that attachment enhances the tolerance of *E. coli* O157:H7 to ferric iron. The attachment also enhanced the expression of the genes *Ecs4441* (3.7), *Ecs1145* (4.7), *Ecs2001* (1.1), *Ecs3393* (3.0) and *Ecs2539* (1.1) related to cold/heat shock, indicating that the attached cells are more resistant to extreme temperatures than planktonic cells. In addition, the genes *Ecs1491* (2.4), *Ecs0165* (1.8) and *Ecs4783* (1.3) related to alkaline pH adaptability were upregulated in attached cells, indicating that the attached cells have increased adaptability to alkaline pH. RT-qPCR results confirmed that attachment enhanced the expression of the stress response genes *rstA*, *rstB*, *cspG*, *cspA*, *htrA*, and *pleD* (Fig. 3d). As shown in Table 2, the concentrations of NADPH and GSH in attached cells increased by 66.2 pmol/L and 11.2 μmol/L, respectively. Figure 4 shows that in a high-pH or ferric iron-containing environment, the survival rate of *E. coli* O157:H7 attached for 1 h is increased by more than double. These results indicate that the attached *E. coli* O157:H7 has enhanced tolerance to general stress, high pH and ferric iron.

**Table 2:** NADPH and GSH concentrations and zeta potentials of quartz-attached and planktonic *E. coli* O157:H7

	NADPH (pmol L-1)	GSH (μmol L-1)
Planktonic cells	505.17 ± 5.9b	53.43 ± 2.2b
Attached cells	571.19 ± 8.4a	64.63 ± 2.6a

Different letters indicate significant differences.

### Antibiotic susceptibility after *E. coli* O157:H7 attachment

**Fig. 5:**
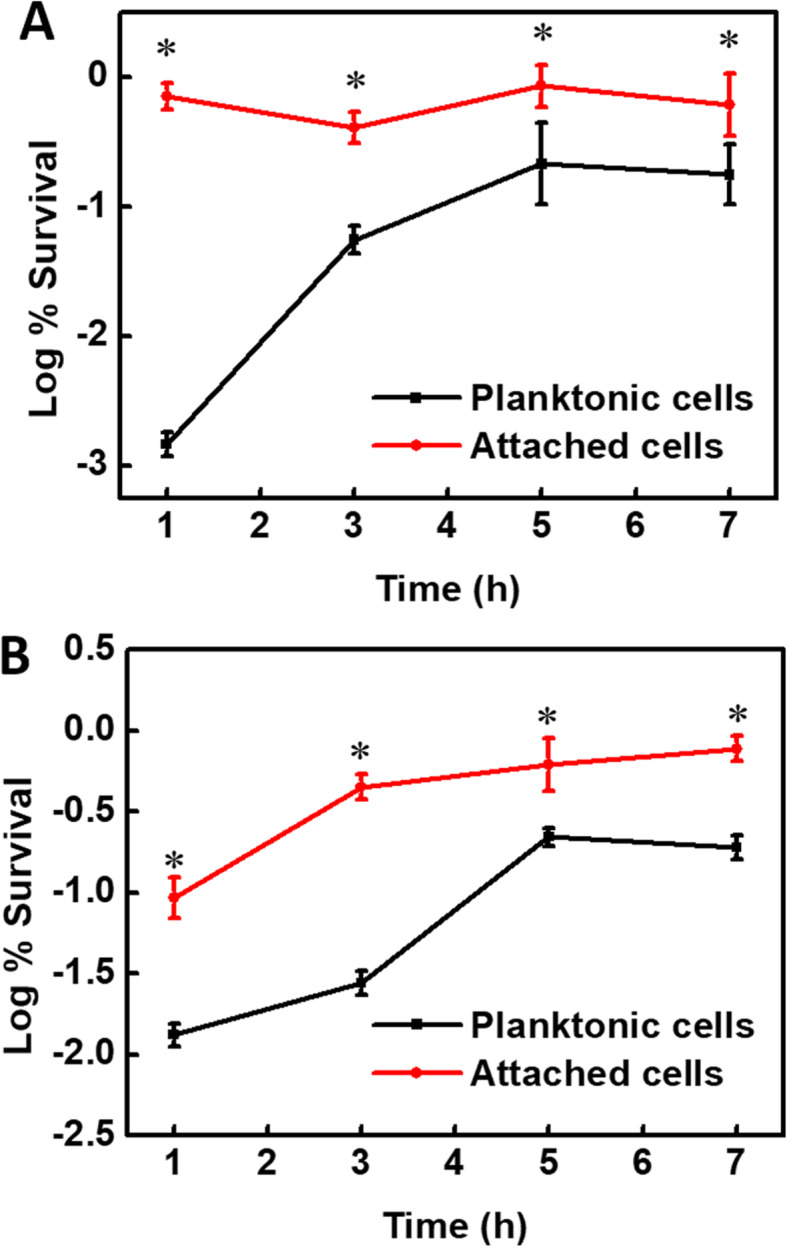
Survival of planktonic and attached cells in the presence of penicillin (**a**) and vancomycin (**b**). Penicillin and vancomycin were applied at 500 μg/mL (dozens of times the MIC of each antibiotic). The attached cells showed reduced antibiotic susceptibility compared to planktonic cells

As shown in Table S6, during attachment, the genes *Ecs0673* (1.5) and *Ecs0135* (1.4) related to β-lactam antibiotic resistance were upregulated, and the gene *Ecs4716* (1.0) related to vancomycin resistance was upregulated. The attachment also increased the expression of the genes *Ecs4425* (2.0), *Ecs1491* (2.4), *ECs0165* (1.8) and *ECs4783* (1.3) related to cationic antimicrobial peptide (CAMP) resistance. The upregulation of these genes was also verified by RT-qPCR analysis (Fig. 3e). As shown in Fig. 5, under penicillin treatment, the survival rate of attached bacteria always exceeded 10%. The survival rate of planktonic bacteria was less than 1% at 1 h, less than 10% at 3 h, and more than 10% at 5–7 h, but it was still significantly lower than that of attached cells. Under vancomycin treatment, the survival rate of attached cells at 1 h was approximately 10% and exceeded 50% at 3–7 h, while the survival rate of planktonic cells at 1–3 h was less than 10% and exceeded 10% at 5–7 h, but it was still significantly lower than that of attached cells. The antibiotic susceptibility of attached *E. coli* O157:H7 cells to penicillin and vancomycin was reduced substantially.

## Discussion

### Functional TCSs promote *E. coli* O157:H7 attachment on quartz

During attachment, the Rcs system promotes bacterial attachment by inhibiting cell metabolism and flagellar assembly and subsequently promotes biofilm formation [13, 25]. The quorum sensing system promotes cell-cell communication by secreting AI-2 to increase cell density and ultimately promotes the attachment of *E. coli* O157:H7 on quartz [7, 14, 15, 26, 27]. The Cpx system promotes attachment by responding to membrane stress and inhibits the production of pili and flagella [11, 17]. Additionally, newly discovered genes were found to affect the attachment of *E. coli* O157:H7, including *baeR*, *baeS*, *barA*, *creC*, *cusS*, *kdpD*, *narQ* and *torS*. Further research is needed to ascertain the role these genes have with regards to the regulatory mechanisms of attachment to surfaces. In general, the attachment of *E. coli* O157:H7 on quartz requires the cooperation of multiple TCSs.

### Process and potential mechanism of *E. coli* O157:H7 attachment on quartz

In *E. coli*, the chemotaxis system regulates the direction of motility, allowing the bacterium to move in the direction of the chemical gradients until it reaches the surface [28, 29]. Therefore, in the planktonic phase, *E. coli* O157:H7 controls the flagella via the chemotaxis system to push the bacteria toward the quartz surface. Once the LPS on the bacterial surface is in contact with the quartz, the surface signal can be transmitted through the Rcs system [30]. The activated Rcs system inhibits *flhD* through *rcsB* (Table 1), which is the top-level controller that controls the fundamental decision of whether to produce flagella, thereby inhibiting the assembly of flagella and promoting stable attachment of *E. coli* O157:H7 on quartz [31, 32]. The flagellum is a key regulatory factor that controls the transition of bacteria from a planktonic state to an attached state [33]. In this study, in the planktonic and attachment stages, the chemotaxis and Rcs systems controlled the motility of flagella, forming a complete attachment regulation process. When the chemotaxis and the Rcs system were damaged, the attachment capacity of *E. coli* O157:H7 was significantly reduced (Fig. 2), further confirming that chemotaxis and the Rcs system are the key systems for regulating the attachment of *E. coli* O157:H7 on quartz.

### Central metabolism shifted toward the fermentative route after attachment

Inhibited fatty acid β-oxidation leads to a decrease in the metabolic activity of *E. coli* O157:H7 on quartz [34]. This is because the attached cells reduce flagellar energy consumption and help *E. coli* O157:H7 survive in a nutrient-poor environment [35]. In addition, the inhibition of arginine and proline metabolism reduces the carbon source in the TCA cycle, resulting in decreased TCA cycle activity [36]. Downregulated TCA cycle activity was also observed for *E. coli* O157:H7 attached to lettuce leaves and soil particles [21, 37]. In addition, damage to flagella leads to increased glycolysis/glycogenesis activity of attached cells [35], and enhanced L-serine metabolism provides energy for *E. coli* O157:H7 in an anaerobic environment [38, 39]. These results indicate that the metabolic pathway of attached *E. coli* O157:H7 is switched from the TCA cycle to the fermentative route. The lower energy consumption prolongs the survival of *E. coli* O157:H7 in the environment and increases the risk of pathogen infection [35].

### Enhanced tolerance to environmental stress in attached cells

The regeneration of NADPH is a cellular response of *E. coli* to oxidative stress [39–41], and the value of GSH represents the ability to resist toxicity challenge and oxidative stress [42]. The increase in NADPH and GSH content in attached cells is beneficial for enhancing the tolerance of *E. coli* O157:H7 to oxidative stress (such as H_2_O_2_) and the degradation of toxic compounds (such as azo dyes) in the environment [23, 35, 43]. In addition, ferric iron and alkaline pH are the main factors that limit the survival of *E. coli* O157:H7 in soil and sediments, respectively [21, 44–46]. Improving the tolerance to ferric iron and alkaline pH can help prolong the survival of *E. coli* O157:H7 in soil and sediment environments, increasing the risk of pathogen infection. In short, attachment is an effective survival strategy for *E. coli* O157:H7, improving the resistance to various environmental stresses and facilitating the survival of the pathogen.

### Reduced antibiotic susceptibility after attachment

Our results demonstrated that before biofilm formation, the antibiotic susceptibility of *E. coli* O157:H7 was reduced upon surface attachment [47–49]. Reduced antibiotic susceptibility promotes the survival of *E. coli* O157:H7 in the medical environment and may increase the production of Shiga toxins, increasing the risk of infection and pathogenicity of the pathogen [23, 50]. In general, attached *E. coli* O157:H7 is more harmful to the host and more difficult to inactivate than planktonic *E. coli*.

## Conclusion

**Fig. 6:**
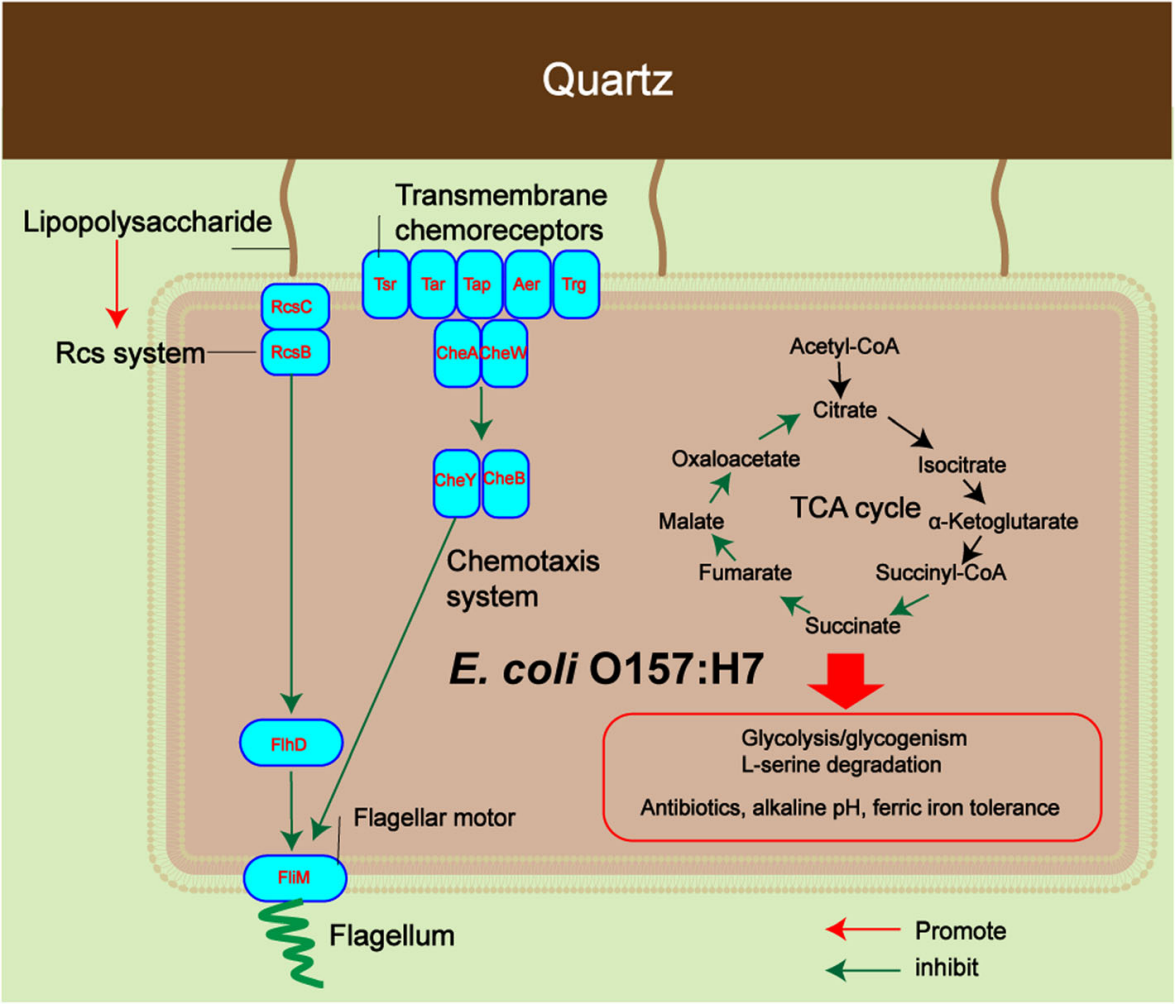
Proposed model for *E. coli* O157:H7 attachment process and physiological properties on quartz. Chemotaxis and Rcs system regulate attachment through flagella. After attachment, the metabolic activity of the bacteria decreases, and the stress resistance increases. This picture refers to the KEGG images [51]

*E. coli* O157:H7 is a global zoonotic pathogen that is responsible for many severe foodborne diseases. After the introduction of pathogens into soil environments by wastewater irrigation, the risk of infection is highly dependent on the interaction of the pathogens with soil particles. The present study is the first to investigate the transcriptomic and proteomic responses of *E. coli* O157:H7 after attachment to mineral surfaces. The attachment of *E. coli* O157:H7 on quartz is regulated by the chemotaxis and Rcs systems. The absence of TCS regulatory genes reduces the attachment of *E. coli* O157:H7 on quartz. After attachment, the metabolic activity of *E. coli* O157:H7 is reduced, the TCA cycle is inhibited, the fermentation metabolism is enhanced, and the tolerance to penicillin, vancomycin, alkaline pH and ferric iron is enhanced (Fig. 6). This knowledge facilitates in-depth understanding of the behavior of bacteria on mineral surfaces, helps combat biofilm formation, and provides new theories for predicting the risk of environmental pathogens.

## Methods

### Bacterial strains and culture conditions

*E. coli* O157:H7 Sakai was provided by the State Key Laboratory of Agricultural Microbiology, Huazhong Agricultural University (Wuhan, China). This bacterium was isolated from soils treated with chicken manure in Fengqiu, Henan Province, China. The bacteria were transferred from a − 80 °C cryopreservation tube to Luria–Bertani (LB) agar plates and incubated at 37 °C for 12 h. A single colony was inoculated into 5 mL of LB medium and cultured at 37 °C for 12 h with shaking at 180 rpm. Then, 1 mL of suspension was inoculated into 100 mL of LB medium and cultured at 37 °C for 12 h with shaking at 180 rpm. The culture was centrifuged (12,000×g for 1 min) and resuspended in sterile LB medium at a final concentration of 1 × 10^9^ cells mL^− 1^.

### Bacterial attachment on quartz

Quartz was purchased from Sinopharm Chemical Reagent Co., Ltd. (Shanghai, China). Quartz particles with a size of approximately 0.5 mm were screened out, washed three times with ddH_2_O, dried, and sterilized at 121 °C for 30 min. The quartz surface was electrically neutral (data not shown). Fifty milliliters of bacterial culture (resuspended cells from section 2.1, 1 × 10^9^ cells mL^− 1^) was mixed with two hundred grams of quartz. Then, the mixed culture was incubated at 37 °C statically to facilitate bacterial attachment. After a 3-h incubation, 1 mL of the upper suspension was aspirated, centrifuged (6000 rpm, 5 min), washed twice with ddH_2_O, and then resuspended with 1 mL of ddH_2_O as planktonic cells. Then, the liquid phase was replaced with 50 mL of ddH_2_O, and the liquid was gently poured out. The liquid phase was replaced with 50 mL of Z-buffer (8.5 g of Na_2_HPO_4_, 5.5 g of NaH_2_PO_4_·H_2_O, 0.75 g of KCl, 0.246 g of MgCl_2_·7H_2_O, ddH_2_O to 1 L, sterilized) [17]. The remaining mixture was vortexed for 30 s to disassociate attached cells from the sand surfaces [17]. The bacteria suspended in Z-buffer were considered attached cells. The collected cells were washed with ddH_2_O and subjected to RNA and protein extraction.

### RNA-seq analysis

RNA-seq analysis was performed as previously described [52]. Briefly, bacterial suspensions were centrifuged at 8000×g for 2 min and resuspended in RNA stabilization reagent (Qiagen, Germany). The cells were lysed by TRIzol, and rRNA was removed via mRNA-ONLY Prokaryotic mRNA Isolation Kit (Epicentre Biotech, Madison, WI, USA). Sequencing was performed by using an Illumina HiSeqTM 2000 with paired-end 100-bp reads (Illumina, San Diego, CA, USA). Qualified reads were mapped to the genome of *Escherichia coli* O157:H7 via SOAP2. The gene expression level was evaluated by the RPKM method (mapped reads per kilobase per million reads). Two biological replicates were used for the RNA-seq experiment. The raw transcriptome data have been deposited in the Sequence Read Archive (SRA: PRJNA511623).

### Protein extraction and reductive alkylation

The collected cells were washed three times with ice-cold phosphate-buffered saline (PBS). The pellets were resuspended in thiourea/urea buffer (7 M urea, 2 M thiourea, 4% w/v CHAPS, 20 mM TBP, and 0.2% Bio-lyte (pH 3–10)) with protease inhibitor and silica beads. The cells were disrupted by vortex mixing and ultrasonication on ice for 10 min. After centrifugation at 12,000×g for 5 min, the Ready Prep 2-D Cleanup Kit (Bio-Rad Laboratories, USA) was used to purify the crude extracts in the supernatant. Reduction and alkylation were performed prior to enzymatic digestion. Proteins were quantified using a 2-D Quant Kit (GE Healthcare, USA). Two biological replicates were used for the proteomics experiment.

### iTRAQ-based quantitative proteomics

iTRAQ analysis was performed as previously described [52, 53]. Briefly, proteins (100 μg) from each sample were subjected to trypsin digestion and labeled with 8-plex iTRAQ reagents (Applied Biosystems, USA). The labeled samples were fractionated using an Ultremex SCX column (250 × 4.6 mm, 5 μm particle size, 200 Å pore size). The eluted samples were then desalted using a Strata X C18 column (Phenomenex, USA), vacuum dried and reconstituted in 2% acetonitrile (ACN) with 0.1% formic acid. Mass spectrometry analysis was performed using a splitless nanoACQuity (Waters, USA) system coupled with a Triple TOF system. The peptides were separated on nanofluidic columns packed with BEH130 C18 (1.7 μm, 100 μm × 100 mm). The solvent gradient conditions, using 2% ACN/0.1% formic acid in water as solvent A and 98% ACN/0.1% formic acid in water as solvent B, were as follows: 5% B from 0 to 1 min, 5–35% B from 1 to 41 min, 35–80% B from 41 to 46 min, and maintained for 5 min. The flow rate was set to a constant value of 300 nL/min.

Data acquisition was carried out using a Triple TOF 5600 System (AB SCIEX, USA) fitted with a Nanospray III source (AB SCIEX, USA) and a pulled quartz tip (New Objectives, USA). The MS was operated with a resolving power equal to or greater than 30,000 FWHM for the TOF MS scans. In information-dependent acquisition (IDA) mode, survey scans were acquired in 250 ms, and as many as 30 product ion scans were collected if they exceeded 120 counts per second with a 2+ to 5+ charge state.

### Protein identification and database search

Protein identification and quantification were carried out using Mascot software (version 2.3.02) as previously described [54, 55]. Searches were performed against the *E. coli* O157:H7 protein database. The search criteria were as follows: i) one missed cleavage by trypsin was allowed; ii) carbamidomethyl cysteine was set as the fixed modification, and methionine was set as the variable modification; iii) the peptide mass tolerance was ±0.05 Da, and the fragment ion tolerance was ±0.1 Da. In the final search results, the false discovery rate (FDR) was less than 1.5%. During protein identification, the significance of comparison had to be lower than 0.05. For protein quantitation, the filters were set as previously described: “median” was chosen for the protein ratio type; the minimum precursor charge was set to 2+, and the minimum peptides were set to 2; only unique peptides were used to quantify proteins [54, 55]. The median intensities were set for normalization, and outliers were removed automatically [54, 55].

### Gene set enrichment analysis (GSEA)

Gene set enrichment analysis (GSEA) was performed as previously described [56]. To overcome the shortcomings of single-gene analysis, gene set enrichment analysis (GSEA) was applied. GSEA annotation was based on GO and KEGG at the transcriptome level and proteomic level, and the data of the two groups were integrated and analyzed, which facilitated the study of gene expression regulation at the coexpression level of the gene set. The significant enrichment analysis of GO functions provides GO terms that are significantly enriched for the differentially expressed protein (or differentially expressed gene) compared to the background of all the identified proteins (or genes) of the species, indicating differentially expressed proteins (or differentially expressed genes) with significantly associated biological functions. The analysis first maps all differentially expressed proteins (or differentially expressed genes) to the various terms of the Gene Ontology database (http://www.geneontology.org), calculates the number of proteins (or genes) for each term, and then applies hypergeometric testing to find GO entries that are significantly enriched for differentially expressed proteins (or differentially expressed genes) compared to the background of all proteins (or genes) of that species. The *P*-value was obtained by hypergeometric calculation, and *P*-value ≤0.05 was used as the threshold. GO terms satisfying this condition were defined as the GO terms that were significantly enriched for the differentially expressed protein (or differentially expressed gene). The main biological functions of differentially expressed proteins (or genes) can be determined by GO significance analysis. The significant pathway enrichment analysis method is the same as the GO function enrichment analysis and is based on the KEGG pathway database. Hypergeometric testing was applied to find pathways that were significantly enriched in differentially expressed proteins (or differentially expressed genes) compared to all identified proteins (or genes) of that species. Determination of significantly enriched pathways can identify the most important biochemical metabolic pathways and signal transduction pathways associated with differentially expressed proteins (or differentially expressed genes).

### Construction of mutant strains

Previous studies have shown that two-component system regulatory genes can affect the attachment of a variety of *E. coli* strains [17]. To verify whether these genes affect the attachment of *E. coli* O157:H7 on the quartz surface, gene knockout mutants were constructed. The target genes and primer pairs are listed in Table S2. First, the helper plasmid pKD46 with an ampicillin resistance fragment was introduced into *E. coli* O157:H7 by electrotransformation. In brief, a single colony of *E. coli* O157:H7 was inoculated into 5 mL of LB medium and cultured at 37 °C for 12 h with shaking at 180 rpm. Then, 50 μL of the suspension was inoculated into 5 mL of fresh LB medium and incubates at 37 °C for 4 h with agitation (180 rpm). When the optical density value (OD_600_) reached 0.5, 1 mL of bacteria was harvested by centrifugation (3000 rpm, 4 °C, 10 min). The supernatant was removed, 1 mL of sterilized distilled deionized water (ddH_2_O) containing 10% glycerol was added at 4 °C to wash the cells, and the cells were then centrifuged (3000 rpm, 4 °C, 10 min) again. This step was repeated 4 times. Then, the cells were resuspended in 40 μL of ddH_2_O containing 10% glycerol. Then, 5 μL of the plasmid pKD46 dissolved in ddH_2_O (Jiangsu Ruiyang Biotechnology Co., Ltd., China) was added to the suspension. The mixture was transferred to a 0.1 cm electrode gap (BIO-RAD, USA). Electric shock was performed at 2.5 kV using an ECM399 electroporator (BTX, USA). The mixture was quickly transferred to 900 μL of LB medium at 37 °C and incubated at this temperature for 1 h with agitation (180 rpm). Then, 100 μL of the solution was applied on an LB plate containing ampicillin (50 mg L^− 1^). The plate was incubated at 30 °C for 12 h, and a single colony (*E. coli* O157:H7- pKD46) was selected. The plasmid pKD46 contains a temperature-controlled replicon and can only be replicated at 30 °C.

Then, *E. coli* O157:H7-pKD46 was used to construct a single gene mutant strain as previously described [57]. Briefly, the gentamicin resistance fragment *dif-Gm-dif* (Jiangsu Ruiyang Biotechnology Co., Ltd., China) containing a repeat sequence (GGTGCGCATAATGTATATTATGTTAAAT) at both ends was inserted into the vector pMD18-T (Figure S2) (TaKaRa, Japan) according to the manufacturer’s instructions. On the recombinant plasmid pMD18-T-*Gm*, one end of the *Gm* fragment contains Hind Ш and Sac I restriction sites, and the other end contains Bam HI and Eco RI restriction sites. PCR was used to amplify fragments upstream and downstream of the target genes. By adding a nucleotide sequence corresponding to the restriction site to the primer, the Hind Ш and Sac I restriction sites were added to the ends of the upstream fragment, and the Bam HI and Eco RI restriction sites were added to the ends of the downstream fragment. The upstream fragment of the target gene was ligated to the vector plasmid using restriction enzymes (Hind Ш and Sac I) and DNA ligase to form the new recombinant plasmid pMD18-T-up-Gm. The downstream fragment of the target gene was ligated into the vector plasmid in the same manner (pMD18-T-up-Gm-down). The fragment up-Gm-down was then excised from the plasmid pMD18-T-up-Gm-down with the restriction enzymes Hind Ш and Eco RI. The fragment up-Gm-down was introduced into *E. coli* O157:H7-pKD46 by electroporation. Transformants were grown at 30 °C on LB plates containing ampicillin and gentamicin (50 mg L^− 1^). Single colonies were selected and serially subcultured at 37 °C for 3 generations to remove the helper plasmid pKD46. PCR was then carried out using primers containing Hind Ш and Eco RI restriction sites. The PCR product was sequenced to check whether the target gene was successfully knocked out.

### Investigation of attachment with a quartz crystal microbalance with dissipation monitoring (QCM-D)

We used an extended QCM technique to measure changes in both the frequency and energy dissipation. Bacterial suspensions (wild type and mutants, Table S2) were added at a final concentration of ~ 10^9^ cells mL^− 1^. Frequency shifts (Δf) and dissipation shifts (ΔD) caused by attachment of the bacteria to the crystal surface were measured continuously for 60 min in filter-sterilized LB medium. The pH of the bacterial cultures was ~ 7.6.

The numbers of attached cells on quartz surfaces were determined after 60 min by direct counting using acridine orange as previously described [17]. Crystals were removed from the QCM chamber and stained for 5 min in a filter-sterilized acridine orange solution [AO 100 μg/mL, 2% (vol/vol) formaldehyde in PBS], and surfaces were examined by epifluorescence microscopy. For each sample, all cells in a minimum of 20 fields of view (80*80 μm in size) were counted.

### Quantitative real-time PCR analyses

Differentially expressed genes in RNA-seq analysis were confirmed by quantitative real-time PCR (qRT-PCR). cDNA was prepared using a reverse transcription kit (HiScript III RT SuperMix for qPCR) (Vazyme, Nanjing, China). The qRT-PCR primers were designed using the online software tools Primer 3 and Beacon Designer 7. The primer sequences are listed in Table S7. qPCR was performed using an RT-PCR system (ABI ViiA™ 7, Thermo Fisher Scientific, USA). The reaction mix was prepared with 5 μL iTaq™ Universal SYBR Green Supermix (BIO-RAD, USA), 2 μL each of the forward and reverse primers, 1 μL of cDNA, and 2 μL of nuclease-free water. The qPCR program for the reaction was 95 °C for 30 s, followed by 40 cycles of 95 °C for 10 s and 55 °C for 20 s, with a final temperature of 60 °C for 35 s. To confirm specific amplification of the PCR products, melting curve analysis was carried out at 95 °C for 5 min and 65 °C for 1 min. The experiment was carried out in 3 biological replicates, and each replicate was analyzed in duplicate. Constitutively expressed *gapA* genes were used as an internal control [58]. A standard graph was plotted for each gene with *gapA* as the endogenous control. The fold change in gene expression was calculated with respect to planktonic cells, and the statistical significance was determined at *p* < 0.05.

### Glutathione (GSH) and nicotinamide adenine dinucleotide phosphate (NADPH) assays

To characterize the antioxidant capacity of the bacteria, we measured the GSH and NADPH concentrations in the bacteria. GSH and NADPH were quantified using the Glutathione Assay Kit (Sigma-Aldrich, USA) and NADP/NADPH Quantification Kit (Sigma-Aldrich, USA) according to the instructions. Samples used in these assays were collected as in the RNA isolation experiments.

### Alkaline pH and ferric iron susceptibility assays

The susceptibility of planktonic and attached cells to alkaline pH and ferric iron was evaluated after 1 h of attachment. We added NaOH (pH 8.0 and 9.0) and FeCl_3_ (1 × 10^− 4^ M) to the attachment system for another one-hour treatment. Before and after the treatment, the number of viable cells was determined by dilution plating on LB plates. The survival rate represents the ratio of the number of viable cells after the treatment to the number of cells before the treatment. Six replicates were performed for this experiment.

### Antibiotic susceptibility assay

To investigate the effects of attachment on *E. coli* O157:H7 antibiotic resistance and to verify changes in antibiotic resistance genes or proteins in transcriptomic and proteomic data, the resistance of planktonic and attached bacteria to penicillin and vancomycin was tested. Fifty milliliters of resuspended overnight culture (1 × 10^9^ cells mL^− 1^) was added to cover 200 g of quartz in glass vials. After 1, 3, 5, and 7 h of static incubation at 37 °C, 500 μg/mL penicillin and vancomycin (dozens of times the MIC of each antibiotic; to kill bacteria within 1 h (data not shown)) were added. After 1 h of treatment, the planktonic and attached bacteria were dissociated as in the attachment experiment. The viability of bacteria in planktonic bacterial suspensions and on quartz was quantified using CFU counting (dilution plating on LB plates). Six biological replicates were performed.

### Statistical analysis

All data are displayed as the mean ± standard deviation (SD). *P*-values were acquired using analysis of variance (ANOVA) followed by Tukey’s multiple comparisons test to evaluate statistical significance using SPSS 17.0 software. Differences were regarded as statistically significant when *p* < 0.05.

## Supplementary Information

**Additional file 1:** **Figure S1.** Differentially expressed genes and proteins during attachment; the y-axis of the graph represents the number of up- and downregulated genes and proteins in attached cells compared to planktonic cells. **Figure S2.** Schematic diagram of the structure of the vector plasmid PMD18-T. **Table S1.** Summary of protein identification in the attached and planktonic *E. coli* O157:H7 using the iTRAQ platform. **Table S2.** Deleted genes and their primers used in this study. **Table S3.** Significantly enriched KEGG pathways in either transcriptomic or proteomic data. **Table S4.** Significantly enriched GO terms in both transcriptomic and proteomic data. **Table S5.** Differentially altered proteins and genes associated with metabolism. **Table S6.** Differentially altered proteins and genes associated with general stress response and antibiotic resistance. **Table S7.** PCR primers used in this study.

## Data Availability

The datasets used and analyzed during the current study are available from the corresponding author on reasonable request. The raw transcriptome data has been deposited in Sequence Read Archive (SRA: PRJNA511623): https://www.ncbi.nlm.nih.gov/bioproject/PRJNA511623.

## References

[1] Atnafie B, Paulos D, Abera M, Tefera G, Hailu D, Kasaye S, et al. Occurrence of *Escherichia coli* O157:H7 in cattle feces and contamination of carcass and various contact surfaces in abattoir and butcher shops of Hawassa, Ethiopia. BMC Microbiol. 2017;(17) ARTN 24. 10.1186/s12866-017-0938-1. 10.1186/s12866-017-0938-1 PMC5264334 28122502

[2] Kisko G, Roller S. Carvacrol and p-cymene inactivate *Escherichia coli* O157 : H7 in apple juice. BMC Microbiol. 2005;5; doi: Artn 36. 10.1186/1471-2180-5-36. 10.1186/1471-2180-5-36 PMC1166557 15963233

[3] Abreham S, Teklu A, Cox E, Tessema TS. *Escherichia coli* O157:H7: distribution, molecular characterization, antimicrobial resistance patterns and source of contamination of sheep and goat carcasses at an export abattoir, Mojdo, Ethiopia. BMC Microbiol. 2019;19(1) ARTN 215. 10.1186/s12866-019-1590-8. 10.1186/s12866-019-1590-8 PMC6740007 31510932

[4] Gagliardi JV, Karns JS (2000). Leaching of Escherichia coli O157: H7 in diverse soils under various agricultural management practices. Appl Environ Microbiol.

[5] Ongeng D, Geeraerd AH, Springael D, Ryckeboer J, Muyanja C, Mauriello G (2015). Fate of Escherichia coli O157:H7 and salmonella enterica in the manure-amended soil-plant ecosystem of fresh vegetable crops: a review. Crit Rev Microbiol.

[6] Jiang XP, Morgan J, Doyle MP (2002). Fate of *Escherichia coli* O157 : H7 in manure-amended soil. Appl Environ Microbiol.

[7] Mauter M, Fait A, Elimelech M, Herzberg M (2013). Surface cell density effects on Escherichia coli gene expression during cell attachment. Environ Sci Technol.

[8] Hong ZN, Jiang J, Li JY, Xu RK, Yan J (2019). Adhesion mediated transport of bacterial pathogens in saturated sands coated by phyllosilicates and Al-oxides. Colloid Surface B.

[9] Wei HZ, Yang G, Wang BY, Li RW, Chen G, Li ZZ (2017). E. coli interactions, adhesion and transport in alumino-silica clays. Colloid Surface B.

[10] Pereira AL, Silva TN, Gomes ACMM, Araujo ACG, Giugliano LG. Diarrhea-associated biofilm formed by enteroaggregative *Escherichia coli* and aggregative Citrobacter freundii: a consortium mediated by putative F pili. BMC Microbiol. 2010;10; doi: Artn 57. 10.1186/1471-2180-10-57. 10.1186/1471-2180-10-57 PMC2836999 20175929

[11] Vogt SL, Raivio TL (2012). Just scratching the surface: an expanding view of the Cpx envelope stress response. FEMS Microbiol Lett.

[12] Vidal O, Longin R, Prigent-Combaret C, Dorel C, Hooreman M, Lejeune P (1998). Isolation of an Escherichia coli K-12 mutant strain able to form biofilms on inert surfaces: involvement of a new ompR allele that increases curli expression. J Bacteriol.

[13] Sharma VK, Bayles DO, Alt DP, Looft T, Brunelle BW, Stasko JA. Disruption of rcsB by a duplicated sequence in a curli-producing *Escherichia coli* O157: H7 results in differential gene expression in relation to biofilm formation, stress responses and metabolism. BMC Microbiol. 2017;17; doi: ARTN 56. 10.1186/s12866-017-0966-x. 10.1186/s12866-017-0966-x PMC5343319 28274217

[14] Lim J, Lee KM, Park CY, Kim HV, Kim Y, Park S (2016). Quorum sensing is crucial to Escherichia coli O157:H7 biofilm formation under static or very slow laminar flow conditions. Biochip J.

[15] Guan YG, Tsao CY, Quan DN, Li Y, Mei L, Zhang JL (2018). An immune magnetic nano-assembly for specifically amplifying intercellular quorum sensing signals. Colloid Surface B.

[16] Prigent-Combaret C, Brombacher E, Vidal O, Ambert A, Lejeune P, Landini P (2001). Complex regulatory network controls initial adhesion and biofilm formation in *Escherichia coli* via regulation of the csgD gene. J Bacteriol.

[17] Otto K, Silhavy TJ (2002). Surface sensing and adhesion of Escherichia coli controlled by the Cpx-signaling pathway. P Natl Acad Sci USA.

[18] Cai P, Huang Q, Walker SL (2013). Deposition and survival of Escherichia coli O157: H7 on clay minerals in a parallel plate flow system. Environ Sci Technol.

[19] Liu X, Zhao W, Huang Q, Cai P (2015). Relative attachment behaviors of pathogenic and nonpathogenic Escherichia coli to soil particles: influence of soil physicochemical properties. Geomicrobiol J.

[20] Oliver DM, Clegg CD, Heathwaite AL, Haygarth PM (2007). Preferential attachment of Escherichia coli to different particle size fractions of an agricultural grassland soil. Water Air Soil Pollut.

[21] Liu X, Gao C, Ji D, Walker SL, Huang Q, Cai P (2017). Survival of Escherichia coli O157: H7 in various soil particles: importance of the attached bacterial phenotype. Biol Fertil Soils.

[22] Cai P, Liu X, Ji DD, Yang SS, Walker SL, Wu YC (2018). Impact of soil clay minerals on growth, biofilm formation, and virulence gene expression of Escherichia coli O157:H7. Environ Pollut.

[23] Landstorfer R, Simon S, Schober S, Keim D, Scherer S, Neuhaus K. Comparison of strand-specific transcriptomes of enterohemorrhagic *Escherichia coli* O157:H7 EDL933 (EHEC) under eleven different environmental conditions including radish sprouts and cattle feces. BMC Genomics. 2014;15; doi: Artn 353. 10.1186/1471-2164-15-353. 10.1186/1471-2164-15-353 PMC4048457 24885796

[24] Bai HJ, Cochet N, Pauss A, Lamy E (2016). Bacteria cell properties and grain size impact on bacteria transport and deposition in porous media. Colloid Surface B.

[25] Oropeza R, Salgado-Bravo R, Calva E (2015). Deletion analysis of RcsC reveals a novel signalling pathway controlling poly-N-acetylglucosamine synthesis and biofilm formation in Escherichia coli. Microbiol-Sgm..

[26] Dewald C, Ludecke C, Firkowska-Boden I, Roth M, Bossert J, Jandt KD (2018). Gold nanoparticle contact point density controls microbial adhesion on gold surfaces. Colloid Surface B.

[27] Vikram A, Jesudhasan PR, Pillai SD, Patil BS. Isolimonic acid interferes with *Escherichia coli* O157:H7 biofilm and TTSS in QseBC and QseA dependent fashion. BMC Microbiol. 2012;12; doi: Artn 261. 10.1186/1471-2180-12-261. 10.1186/1471-2180-12-261 PMC3562146 23153211

[28] Sourjik V, Armitage JP (2010). Spatial organization in bacterial chemotaxis. EMBO J.

[29] Porter SL, Wadhams GH, Armitage JP (2011). Signal processing in complex chemotaxis pathways. Nat Rev Microbiol.

[30] Morgenstein RM, Rather PN (2012). Role of the Umo proteins and the Rcs Phosphorelay in the swarming motility of the wild type and an O-antigen (waaL) mutant of Proteus mirabilis. J Bacteriol.

[31] Francez-Charlot A, Laugel B, Van Gemert A, Dubarry N, Wiorowski F, Castanie-Cornet MP (2003). RcsCDB his-asp phosphorelay system negatively regulates the flhDC operon in Escherichia coli. Mol Microbiol.

[32] Wang SY, Fleming RT, Westbrook EM, Matsumura P, McKay DB (2006). Structure of the Escherichia coli FlhDC complex, a prokaryotic heteromeric regulator of transcription. J Mol Biol.

[33] Belas R (2014). Biofilms, flagella, and mechanosensing of surfaces by bacteria. Trends Microbiol.

[34] Polyak SW, Abell AD, Wilce MCJ, Zhang L, Booker GW (2012). Structure, function and selective inhibition of bacterial acetyl-coa carboxylase. Appl Microbiol Biotechnol.

[35] Martinez-Garcia E, Nikel PI, Chavarria M, de Lorenzo V (2014). The metabolic cost of flagellar motion in Pseudomonas putida KT2440. Environ Microbiol.

[36] Nishida I, Watanabe D, Takagi H (2016). Putative mitochondrial alpha-ketoglutarate-dependent dioxygenase Fmp12 controls utilization of proline as an energy source in *Saccharomyces cerevisiae*. Microbial Cell.

[37] Fink RC, Black EP, Hou Z, Sugawara M, Sadowsky MJ, Diez-Gonzalez F (2012). Transcriptional responses of Escherichia coli K-12 and O157:H7 associated with lettuce leaves. Appl Environ Microbiol.

[38] Lim SJ, Jung YM, Shin HD, Lee YH (2002). Amplification of the NADPH-related genes zwf and gnd for the oddball biosynthesis of PHB in an E-coli transformant harboring a cloned phbCAB operon. J Biosci Bioeng.

[39] Zhao F, Wang YT, An HR, Hao YL, Hu XS, Liao XJ. New Insights into the Formation of Viable but Nonculturable *Escherichia coli* O157:H7 Induced by High-Pressure CO2. Mbio. 2016;7(4) ARTN e00961. 10.1128/mBio.00961-16. 10.1128/mBio.00961-16 PMC4999544 27578754

[40] Mols M, van Kranenburg R, Tempelaars MH, van Schaik W, Moezelaar R, Abee T (2010). Comparative analysis of transcriptional and physiological responses of Bacillus cereus to organic and inorganic acid shocks. Int J Food Microbiol.

[41] Aertsen A, De Spiegeleer P, Vanoirbeek K, Lavilla M, Michiels CW (2005). Induction of oxidative stress by high hydrostatic pressure in Escherichia coli. Appl Environ Microbiol.

[42] Yuan C, Fu X, Huang L, Ma Y, Ding X, Zhu L (2016). The synergistic antiviral effects of GSH in combination with acyclovir against BoHV-1 infection in vitro. Acta Virol.

[43] Lee H, Lee DG (2018). Gold nanoparticles induce a reactive oxygen species-independent apoptotic pathway in Escherichia coli. Colloid Surface B.

[44] Zhang TX, Hu SP, Yang WH (2017). Variations of *Escherichia coli* O157:H7 Survival in Purple Soils. Int J Environ Res Public Health.

[45] Yao ZY, Yang L, Wang HZ, Wu JJ, Xu JM (2015). Fate of Escherichia coli O157: H7 in agricultural soils amended with different organic fertilizers. J Hazard Mater.

[46] Liang CL, Yao ZY, Du SC, Hong M, Wang K, Zhang DM (2019). Sediment pH, not the bacterial diversity, determines Escherichia coli O157:H7 survival in estuarine sediments. Environ Pollut.

[47] Fu YZ, Deering AJ, Bhunia AK, Yao Y (2017). Biofilm of Escherichia coli O157:H7 on cantaloupe surface is resistant to lauroyl arginate ethyl and sodium hypochlorite. Int J Food Microbiol.

[48] Kim NH, Rhee MS (2016). Synergistic bactericidal action of phytic acid and sodium chloride against Escherichia coli O157:H7 cells protected by a biofilm. Int J Food Microbiol.

[49] Scotti R, Nicolini L, Stringaro A, Gabbianelli R (2015). A study on prophagic and chromosomal sodC genes involvement in Escherichia coli O157:H7 biofilm formation and biofilm resistance to H2O2. Ann I Super Sanita.

[50] Khan ST, Musarrat J, Al-Khedhairy AA (2016). Countering drug resistance, infectious diseases, and sepsis using metal and metal oxides nanoparticles: current status. Colloid Surface B.

[51] Kanehisa M (2019). Toward understanding the origin and evolution of cellular organisms. Protein Sci.

[52] Wang SH, You ZY, Ye LP, Che JQ, Qian QJ, Nanjo YH (2014). Quantitative proteomic and Transcriptomic analyses of molecular mechanisms associated with low silk production in silkworm Bombyx mori. J Proteome Res.

[53] Trevisan S, Manoli A, Ravazzolo L, Botton A, Pivato M, Masi A (2015). Nitrate sensing by the maize root apex transition zone: a merged transcriptomic and proteomic survey. J Exp Bot.

[54] Wang JP, Mei H, Zheng C, Qian HL, Cui C, Fu Y (2013). The metabolic regulation of sporulation and Parasporal crystal formation in bacillus thuringiensis revealed by Transcriptomics and proteomics. Mol Cell Proteomics.

[55] Chen Z, Wen B, Wang QH, Tong W, Guo J, Bai X (2013). Quantitative proteomics reveals the temperature-dependent proteins encoded by a series of cluster genes in Thermoanaerobacter Tengcongensis. Mol Cell Proteomics.

[56] Croken MM, Qiu WG, White MW, Kim K. Gene Set Enrichment Analysis (GSEA) of Toxoplasma gondii expression datasets links cell cycle progression and the bradyzoite developmental program. BMC Genomics. 2014;15; doi: Artn 515. 10.1186/1471-2164-15-515. 10.1186/1471-2164-15-515 PMC4092224 24962434

[57] Yamamoto N, Nakahigashi K, Nakamichi T, Yoshino M, Takai Y, Touda Y, et al. Update on the Keio collection of *Escherichia coli* single-gene deletion mutants. Mol Syst Biol. 2009;(5) ARTN 335. 10.1038/msb.2009.92. 10.1038/msb.2009.92 PMC2824493 20029369

[58] Fitzmaurice J, Glennon M, Duffy G, Sheridan JJ, Carroll C, Maher M (2004). Application of real-time PCR and RT-PCR assays for the detection and quantitation of VT 1 and VT 2 toxin genes in E-coli O157 : H7. Mol Cell Probes.

