# Supplementary material for: The attachment process and physiological properties of Escherichia coli O157:H7 on quartz
Source: BMC Microbiol. 2020 Nov 19;20:355. doi: 10.1186/s12866-020-02043-8 (PMC7677791; doi:10.1186/s12866-020-02043-8)
Supplement: Supplementary file 1 — Additional file 1: Figure S1. Differentially expressed genes and proteins during attachment; the y-axis of the graph represents the number of up- and downregulated genes and proteins in attached cells compared to planktonic cells. Figure S2. Schematic diagram of the structure of the vector plasmid PMD18-T. Table S1. Summary of protein identification in the attached and planktonic E. coli O157:H7 using the iTRAQ platform. Table S2. Deleted genes and their primers used in this study. Table S3. Significantly enriched KEGG pathways in either transcriptomic or proteomic data. Table S4. Significantly enriched GO terms in both transcriptomic and proteomic data. Table S5. Differentially altered proteins and genes associated with metabolism. Table S6. Differentially altered proteins and genes associated with general stress response and antibiotic resistance. Table S7. PCR primers used in this study. [file 12866_2020_2043_MOESM1_ESM.docx]

**The attachment process and physiological properties of *Escherichia coli* O157:H7 on quartz**

Liliang Wang, Yichao Wu, Peng Cai^*^, Qiaoyun Huang

State Key Laboratory of Agricultural Microbiology, College of Resources and Environment, Huazhong Agricultural University, Wuhan 430070, China

*Corresponding author: Peng Cai

State Key Laboratory of Agricultural Microbiology

Huazhong Agricultural University, Wuhan, China

Phone: +86 27 87671033; Fax: +86 27 87280670

E-mail address: cp@mail.hzau.edu.cn


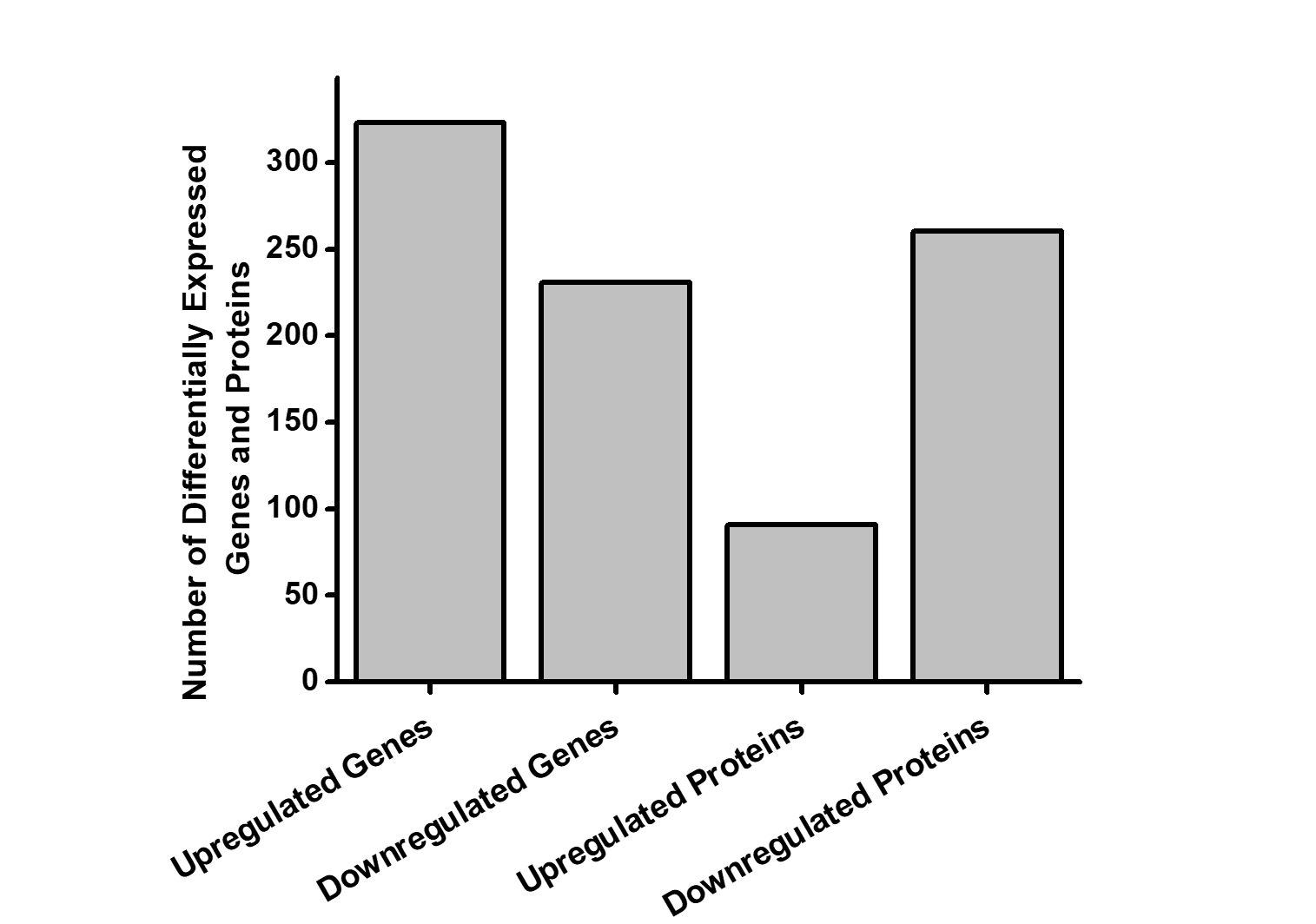


**Figure S1.** Differentially expressed genes and proteins during attachment, the y-axis in the graph represent the number of up- and downregulated genes and proteins in attached cells compared to planktonic cells.


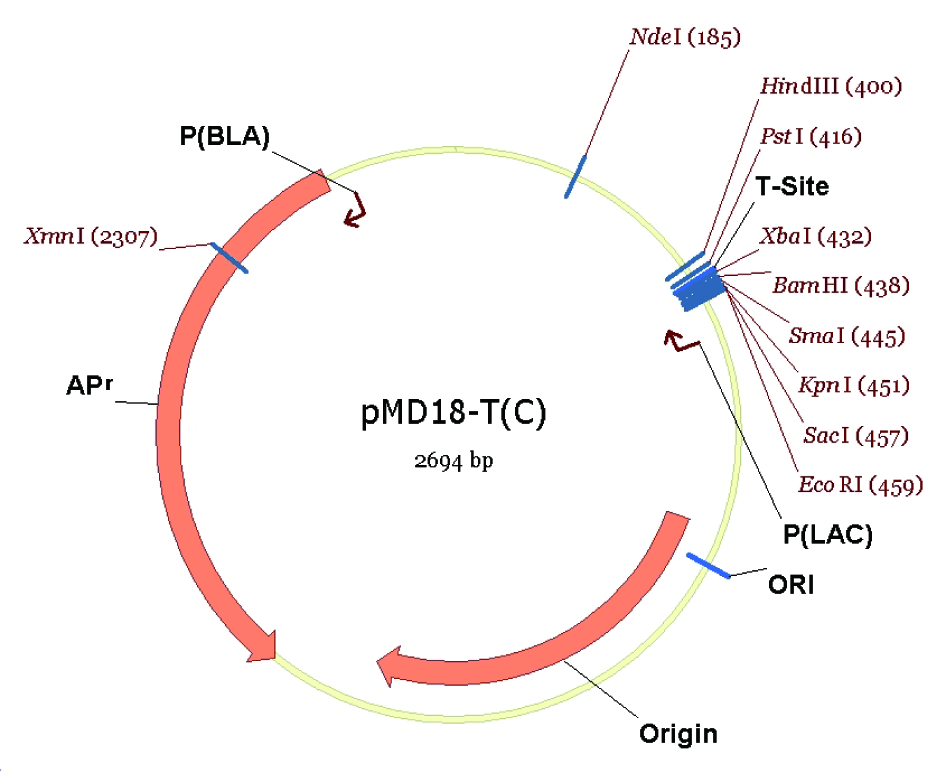


**Figure S2.** Schematic diagram of the structure of the vector plasmid PMD18-T.

**Table S1.** Summary of protein identification in the attached and planktonic *E. coli* O157:H7 using the iTRAQ platform.

| Category | Identified number |
| --- | --- |
| Total spectra | 372871 |
| Spectra | 167805 |
| Unique Spectra | 164972 |
| Peptide | 18419 |
| Unique Peptide | 18157 |
| Protein | 2782 |

**Table S2**. Deleted genes and their primers used in this study.

| Target genes | Gene description | Fragment site | Restriction enzyme cutting site | Sequence |
| --- | --- | --- | --- | --- |
| *baeS* | Modification of lipopolysaccharides | up | Hind III | Forward:CCCAAGCTT TTTTGAGCGTGGCTTTATT |
|  |  |  | Sal I | Reverse:ACGCGTCGAC AGAATGGGTCGCCGTGTA |
|  |  | down | BamHI | Forward:CGGGATCC CGTCTTTACAGGCGGAGGT |
|  |  |  | EcoRI | Reverse:GGAATTC CACTGTCGGCAAAGGTCA |
| *baeR* | Modification of lipopolysaccharides | up | Hind III | Forward:CCCAAGCTT ATCGTGGAAGATGAACCG |
|  |  |  | Sal I | Reverse:ACGCGTCGAC CGCCAATCTCCAGCCCTA |
|  |  | down | BamHI | Forward:CGGGATCC GCTGAAAGCCCGTTGATT |
|  |  |  | EcoRI | Reverse:GGAATTC GGTAACCGACGCCGTAAA |
| *qseB* | Quorum sensing | up | Hind III | Forward:CCCAAGCTT CACAAGGTCGTCAGGGAA |
|  |  |  | Sal I | Reverse:ACGCGTCGAC GACGCAGCCCTTCTACAC |
|  |  | down | BamHI | Forward:CGGGATCC ACGCTGGCTGGCGAACCCTT |
|  |  |  | EcoRI | Reverse:GGAATTC CAACGGCATTACTGGTGA |
| *kdpD* | Potassium transport | up | Hind III | Forward:CCCAAGCTT TCCGCAAAGGCAATCTGA |
|  |  |  | Sal I | Reverse:ACGCGTCGAC TGTACCCGCCACTTGTCTT |
|  |  | down | BamHI | Forward:CGGGATCC TGGTGATCGGGAACCTTACT |
|  |  |  | EcoRI | Reverse:GGAATTC GTAAGCGGAGTGCGTAAATC |
| *torS* | Trimethylamine N-oxide respiration | up | Hind III | Forward:CCCAAGCTT CTGACCAGTGCCGATAAC |
|  |  |  | Sal I | Reverse:ACGCGTCGAC GACTGACTTCGCTGCTAAACT |
|  |  | down | BamHI | Forward:CGGGATCC GAAGCAGGTGGCAAGAATG |
|  |  |  | EcoRI | Reverse:GGAATTC TCTTCATTGAGTTGCGATACA |
| *creC* | Carbon source-responsive | up | Hind III | Forward:CCCAAGCTT GTGCGAAGAGCAACGGAGGG |
|  |  |  | Sal I | Reverse:ACGCGTCGAC GGCAACATACATCACCGAACT |
|  |  | down | BamHI | Forward:CGGGATCC CCTGAACTCGGCAGTAGCG |
|  |  |  | EcoRI | Reverse:GGAATTC TTATGCGACCGCTCTTGG |
| *cusS* | Copper or silver efflux | up | Hind III | Forward:CCCAAGCTT GGTCAGTAAGCCATTTCAGC |
|  |  |  | Sal I | Reverse:ACGCGTCGAC GAACCGCCAATAGCACGA |
|  |  | down | BamHI | Forward:CGGGATCC AACGGAAATCGCCCTCAG |
|  |  |  | EcoRI | Reverse:GGAATTC CGGTAGAAACGGTCAAACA |
| *tar* | Aspartate sensor receptor | up | Hind III | Forward:CCCAAGCTT GATGTAATAGCGTTGAGA |
|  |  |  | Sal I | Reverse:ACGCGTCGAC GCTGGCGAATGGTTTGTA |
|  |  | down | BamHI | Forward:CGGGATCC GCAGATAGCGGGGAGAAC |
|  |  |  | EcoRI | Reverse:GGAATTC AAAAGCACAGGGTTGATG |
| *cheA* | Chemotaxis protein, sensor kinase | up | Hind III | Forward:CCCAAGCTT TAATAGTGAAAGCCCAAT |
|  |  |  | Sal I | Reverse:ACGCGTCGAC ATAATGTCGGTGTTGAGT |
|  |  | down | BamHI | Forward:CGGGATCC CCGCTGAATGCTGTTATG |
|  |  |  | EcoRI | Reverse:GGAATTC CGTCACGGCAAACTCTGG |
| *narQ* | Nitrogen metabolism | up | Hind III | Forward:CCCAAGCTT GCCACGGTTGATGTTCCT |
|  |  |  | Sal I | Reverse:ACGCGTCGAC CTGAGCCAACACCATTTT |
|  |  | down | BamHI | Forward:CGGGATCC TGCCTCCTACTGACCAAA |
|  |  |  | EcoRI | Reverse:GGAATTC AAGGCAATGGTCAGAATG |
| *rcsC* | Biofilm formation | up | Hind III | Forward:CCCAAGCTT ATGGGCAAAGCCGACAAA |
|  |  |  | Sal I | Reverse:ACGCGTCGAC GTTACCGCAATGTTCTGTC |
|  |  | down | BamHI | Forward:CGGGATCC ATCGTAGGACGGATAAGG |
|  |  |  | EcoRI | Reverse:GGAATTC ATCGTGCTGAAACAAGGTG |
| *barA* | Carbon storage regulator | up | Hind III | Forward:CCCAAGCTT TTTGCTTCGCTGCTGTAA |
|  |  |  | Sal I | Reverse:ACGCGTCGAC CAAAACGACGCACGACGA |
|  |  | down | BamHI | Forward:CGGGATCC TTTTATGACGCTGTTTTC |
|  |  |  | EcoRI | Reverse:GGAATTC ATTTCCCTGGCGATGTTT |
| *cpxA* | Cell envelope protein folding | up | Hind III | Forward: CCCAAGCTT TTCTGGACGACAGCATTG |
|  |  |  | Sal I | Reverse:ACGCGTCGAC CCACGGGTGACCATCTTT |
|  |  | down | BamHI | Forward:CGGGATCC ACTCCGCATTTGTAGGCA |
|  |  |  | EcoRI | Reverse:GGAATTC GACGGTCTCACGGAAAGTA |

**Table S3** Significantly enriched KEGG pathways in either transcriptomic or proteomic data.

|  | Attached/Planktonic | | Gene or protein description |
| --- | --- | --- | --- |
|  | Protein | Gene ID |  |
| Arginine and proline metabolism | AstB (-0.6) |  | N-succinylarginine dihydrolase |
|  | AstA (-0.1) |  | Arginine N-succinyltransferase |
|  | PuuA (-1.0) |  | Glutamate--putrescine ligase |
|  | PuuB (-1.3) |  | Gamma-glutamylputrescine oxidoreductase |
|  | PuuC (-0.9) |  | Aldehyde dehydrogenase |
|  | SpeG (-0.6) |  | Spermidine N(1)-acetyltransferase |
|  | PatA (-0.8) |  | Putrescine aminotransferase |
|  | SpeE (-0.3) |  | Polyamine aminopropyltransferase |
|  | LysA (-0.6) |  | Diaminopimelate decarboxylase |
|  | XthA (-0.5) |  | Exonuclease III |
|  |  |  |  |
| Bacterial chemotaxis | Tsr (-0.5) |  | Methyl-accepting chemotaxis protein I, serine sensor receptor |
|  | Tar (-0.6) |  | Methyl-accepting chemotaxis protein II, aspartate sensor receptor |
|  | DppA (-0.6) |  | Dipeptide/heme ABC transporter periplasmic binding protein |
|  | Tap (-0.7) |  | Methyl-accepting chemotaxis protein IV |
|  | Aer (-0.9) |  | Aerotaxis receptor |
|  | CheB (-0.5) |  | Protein-glutamate methylesterase/protein-glutamine glutaminase |
|  | CheA (-0.8) |  | Chemotaxis protein |
|  | CheW (-0.4) |  | Chemotaxis protein |
|  |  |  |  |
| Ribosome | RplR (-0.7) |  | 50S ribosomal protein L18 |
|  | RpsU (-0.5) |  | 30S ribosomal protein S21 |
|  | SpeE (-0.3) |  | Polyamine aminopropyltransferase |
|  | RpsL (-0.8) |  | 30S ribosomal protein S12 |
|  | RpmG (-0.7) |  | 50S ribosomal protein L33 |
|  | RpsR (-0.2) |  | 30S ribosomal protein S18 |
|  | DadX (-0.7) |  | Alanine racemase, catabolic |
|  | RpmH (0.3) |  | 50S ribosomal protein L34 |
|  | RplT (0.3) |  | 50S ribosomal protein L20 |
|  | RpmJ (-1.3) |  | 50S ribosomal protein L36 |
|  | RpmD (0.4) |  | 50S ribosomal protein L30 |
|  | NagA (0.4) |  | N-acetylglucosamine-6-phosphate deacetylase |
|  | RpmF (-0.8) |  | 50S ribosomal protein L32 |
|  | RplO (-0.5) |  | 50S ribosomal protein L15 |
|  | RpmI (-1.1) |  | 50S ribosomal protein L35 |
|  | RpsN (-0.6) |  | 30S ribosomal protein S14 |
|  | RplW (-1.0) |  | 50S ribosomal protein L23 |
|  | RpsT (-0.8) |  | 30S ribosomal protein S20 |

**Table S4** Significantly enriched GO terms in both transcriptomic and proteomic data.

|  | Attached/Planktonic | | Gene or protein description |
| --- | --- | --- | --- |
|  | Protein | Gene ID |  |
| Fatty acid beta-oxidation | FadM (-0.4) |  | Long-chain acyl-CoA thioesterase III |
|  | FadB (-0.6) | *ECs4774* (-2.5) | Fatty acid oxidation complex subunit alpha |
|  | FadE (-0.7) | *ECs0248* (-1.9) | Acyl-coenzyme A dehydrogenase |
|  | FadA (-0.6) | *ECs4773* (-2.9) | 3-ketoacyl-CoA thiolase |
|  |  | *ECs3225* (-1.5) | 3-ketoacyl-CoA thiolase |
|  |  | *ECs3224* (-1.9) | Multifunctional fatty acid oxidation complex subunit alpha |
|  |  | *ECs2514* (-1.8) | Long-chain-fatty-acid--CoA ligase |
|  |  |  |  |
| Iron ion binding | FepA (-0.7) | *ECs0623* (2.0) | Ferrienterobactin outer membrane transporter |
|  | IscU (-0.8) | *ECs3395* (2.7) | Iron-sulfur cluster assembly scaffold protein |
|  | IscA (-0.5) | *ECs3394* (2.3) | Iron-binding protein |
|  | NfuA (-0.4) | *ECs4256* (1.7) | Fe/S biogenesis protein |
|  | IscR (-0.6) | *ECs3397* (2.8) | HTH-type transcriptional regulator |
|  | CydB (-0.8) | *ECs0769* (-2.1) | Cytochrome bd-I ubiquinol oxidase subunit 2 |
|  | CyoB (-0.6) |  | Cytochrome bo(3) ubiquinol oxidase subunit 1 |
|  | YeaW (-0.9) |  | Carnitine monooxygenase oxygenase subunit |
|  | CybC (-0.9) |  | Soluble cytochrome b562 |
|  | RlmN (-0.3) |  | Dual-specificity RNA methyltransferase |
|  | TorY (1.4) |  | Cytochrome c-type protein |
|  |  | *ECs0154* (1.2) | Ferrichrome outer membrane transporter |
|  |  | *ECs3390* (1.8) | Hypothetical protein |
|  |  | *ECs1480* (3.2) | Ferric-rhodotorulic acid outer membrane transporter |
|  |  | *ECs4152* (-1.3) | Peptide deformylase |
|  |  | *ECs4189* (2.4) | Bacterioferritin |
|  |  | *ECs3920* (-1.0) | Cyclic 3',5'-adenosine monophosphate phosphodiesterase |
|  |  | *ECs3619* (3.3) | Sulfite reductase subunit alpha |
|  |  | *ECs5154* (1.4) | Transcriptional repressor NsrR |
|  |  | *ECs2487* (-2.0) | Methionine sulfoxide reductase B |
|  |  | *ECs3165* (-1.7) | NADH dehydrogenase subunit I |
|  |  | *ECs2613* (-2.0) | Ferritin |
|  |  | *ECs2365* (-2.3) | Superoxide dismutase |
|  |  | *ECs5052* (-2.4) | Cytochrome c552 |
|  |  | *ECs0890* (-1.4) | DNA starvation/stationary phase protection protein Dps |
|  |  | *ECs3650* (-1.2) | Flavodoxin |
|  |  | *ECs3171* (-1.4) | NADH dehydrogenase subunit B |

**Table S5** Differentially altered proteins and genes associated with metabolism.

|  | Attached/Planktonic | | Gene or protein description |
| --- | --- | --- | --- |
|  | Protein | Gene ID |  |
| Pyruvate metabolism |  | *ECs4245* (-2.3) | Phosphoenolpyruvate carboxykinase |
|  | AceB (-0.4) |  | Malate synthase |
|  |  |  |  |
| Citrate cycle (TCA cycle) |  | *ECs4245* (-2.3) | Phosphoenolpyruvate carboxykinase |
|  |  | *ECs0754* (-1.0) | Succinyl-CoA synthetase subunit alpha |
|  |  | *ECs0748* (-1.1) | Succinate dehydrogenase flavoprotein subunit, SdhA |
|  |  | *ECs0749* (-1.1) | Succinate dehydrogenase iron-sulfur subunit, SdhB |
|  |  | *ECs2317* (-1.8) | Fumarate hydratase |
|  |  | *ECs2318* (-1.7) | Fumarase A |
|  |  | *ECs5132* (-2.4) | Fumarate reductase |
|  |  | *ECs4109* (-1.0) | Malate dehydrogenase |
|  |  | *ECs4673* (-2.3) | ATP synthase subunit epsilon |
|  | FrdD (-0.9) |  | Fumarate reductase subunit D |
|  | Mdh (-0.7) |  | Malate dehydrogenase |
|  |  |  |  |
| Glycolysis/Gluconeogenesis | Pgk (1.3) |  | Phosphoglycerate kinase |
|  | Glk (1.3) |  | Glucokinase |
|  |  |  |  |
| L-serine metabolism |  | *ECs2523* (1.4) | L-serine dehydratase |
|  |  | *ECs3784* (1.9) | L-serine biosynthetic |
|  |  | *ECs0990* (1.3) | L-serine biosynthetic |

**Table S6** Differentially altered proteins and genes associated with general stress response and antibiotic resistance.

|  | Attached/Planktonic | | Gene or protein description |
| --- | --- | --- | --- |
|  | Protein | Gene ID |  |
| Stress response |  | *ECs2315*（1.4） | Sensor histidine kinase RstB |
|  |  | *ECs2314*（2.0） | DNA-binding transcriptional regulator RstA |
|  |  | *ECs0165* (1.8) | Cell envelope protein folding, and protein degradation |
|  |  | *ECs0623* (2.0) | Ferric enterobactin receptor PfeA |
|  |  |  |  |
| Cold or heat shock |  | *ECs4441* (3.7) | Cold shock protein (beta-ribbon, CspA family) |
|  |  | *ECs1145* (4.7) | Cold shock protein (beta-ribbon, CspA family) |
|  |  | *ECs2001* (1.1) | Heat shock protein HslJ |
|  |  | *ECs3393* (3.0) | Heat shock protein binding, cochaperone HscB |
|  |  | *ECs2539* (1.1) | Heat shock protein HtpX |
|  |  |  |  |
| RpoS system |  | *ECs1737* (1.6) | Response regulator of RpoS |
|  |  |  |  |
| Acidic pH |  | *ECs4425* (2.0) | Phosphoethanolamine transferase |
|  |  |  |  |
| Alkaline pH |  | *ECs1491* (2.4) | L,D-transpeptidase YcfS |
|  |  | *ECs0165* (1.8) | Serine endoprotease DegP |
|  |  | *ECs4783* (1.3) | Thiol:disulfide interchange protein DsbA |
|  |  |  |  |
| Antibiotic resistance |  | *ECs0673* (1.5) | Penicillin-binding protein 2 |
|  |  | *ECs0135* (1.4) | Aspartate decarboxylase |
|  |  | *ECs4716* (1.0) | Transcription termination factor，Rho |
|  |  |  |  |
| Cationic antimicrobial peptide (CAMP) resistance | | *ECs4425* (2.0) | KDO II ethanolamine phospho transferase, EptB |
|  |  | *ECs1491* (2.4) | L,D-transpeptidase YcfS |
|  |  | *ECs0165* (1.8) | Serine protease Do, DegP |
|  |  | *ECs4783* (1.3) | Thiol:disulfide interchange protein DsbA |

**Table S7**. PCR primers used in this study.

| Target genes | Primers sequence（5'-3'） | Target genes | Primers sequence（5'-3'） |
| --- | --- | --- | --- |
| Internal control |  | TCA cycle |  |
| *gapA* | GGGACGAAGTTGGTGTTGAC | ECs4245 | GTACGCAGAAGTGCTGGTGA |
|  | AACCACTTTCTTCGCACCAG |  | GGCAGAGTGAAGGTTTCTGC |
|  |  | ECs0754 | TGCTGACCGTGAAAGTGAAG |
| Glycolysis |  |  | CGGTTTGTGAATGTGACCAG |
| *pstC* | CTGGCAGCCATTCCAAGTAT | *sdhA* | CCAGCGAGTCTGATGTTGAA |
|  | GCGAGGATACCGATACCAAA |  | TTCAACTGCTCAAGCCCTTT |
| ECs4664 | TGAAGTCTGGTGAGCTGGTG | *sdhB* | GGCCTGTATTACCCCGATTT |
|  | GCTGGTGAAGACGAAGGAAG |  | TGGCGGATTTTGTCCATTAT |
| ECs2907 | GAAGAGGCCAGTCAGTTTGC | *fumC* | CATTACCTGTGCACCGTTTG |
|  | TCCAGGGTGTTTGAGAGCTT |  | CTCATTTTCCGGGATTGAGA |
| ECs2846 | TGCAGGGATCAAAGAAATCC | ECs2318 | GTTGTCGCAGTATCCCGTTT |
|  | ACGCACGTTCATAATGGTCA |  | AGAACCGGAGGCATAACCTT |
| *pgk* | GGCACTGAAAGAACCTGCTC | ECs4109 | GGATCGTTCCGACCTGTTTA |
|  | TAGCGATACCACCACCAACA |  | GGTAACGCCGAACAGTTTGT |
|  |  |  |  |
| Amino acid transport and metabolism | | Bacterial infections | |
| ECs2523 | TAATGCCATTGCCTCTGTGA | *eae* | ACGGTCTGGATCGTATCGTC |
|  | CATGTCCTTACCGGTTTCGT |  | CTTGCACATAAGCAGGCAAA |
| ECs3784 | GCGATCCATTTACCTCTCCA | *ospE* | CACTTGGCATCAACCTGAAA |
|  | AGAGAGCGTTGAGCCATTGT |  | GGGAGGACGCATATCAAAAC |
| ECs0990 | CGTGAATGGCAACTCTCTGA | *tpgB2* | CTTGATGTTAAAGCGGCACA |
|  | CGGGAAAGAATGGTTGAAGA |  | TCTGCAAGCACGTTTGCTAT |
|  |  |  |  |
| Bacterial chemotaxis |  | Cationic antimicrobial peptide (CAMP) resistance | |
| *rbsB* | CCGGATGTTCAGGCTGTATT | *arnB* | TATTATCGCGAGCGTTTTCC |
|  | CGGGTAGCTGAGCGATAGTC |  | TATTGTCCTGCGAGTTGCTG |
| *mglB* | TGTGATGGCCAGCATGTTAT | *arnT* | TTTCGCTATGGGCATTTTTC |
|  | TGATCGTTCTGCTTGGACTG |  | TATAGCGGCTTGGCTGAAGT |
| *tap* | AAGGGCTGATCGAAGAGTCA | *degP* | CTTGGTCTGGTGGCAGGTAT |
|  | CCGAAGCAATTTCTCCCATA |  | CCAGAGACCAGGTGGGAATA |
| *aer* | GGCACCATTACTTCGCTGAT | *dsbA* | GTGACTGTTCCGCTGTTTGA |
|  | TCAGCTTGCGAATATCGTTG |  | GAGCGACCAGAGATTTCACC |
|  |  |  |  |
| General stress response |  | Flagellar assembly | |
| *cspG* | GGTTTTGGCTTTATCACTCCTG | *flgE* | ACAGGCCAGTAACCTGATCG |
|  | CACGTTGACCCTGCTCAATA |  | GATGGTGGCCGTAACTTTGT |
| *cspA* | TTCATCACTCCTGACGATGG | *flgB* | TGACCTCAACGCAACACATT |
|  | TTACAGGCTGGTTACGTTGC |  | CAGGCTGTTATCGGCAAACT |
| *rstB* | CGTGCTGGATAATTTGCTCA | *fliC* | TCAGCTTCAAAACGTGATGC |
|  | ATATGTTCGCGGTTTTCTGG |  | CAAGTTGCCTGCATCGTCTA |
| *rstA* | GTGGAAGATGATGCGGAAGT | *flgC* | AAAATGCCGAACGTTGATGT |
|  | CGCGTAAATCACGACAAATG |  | GCGTAAGGGTTTTCAGCATC |
| *htrA* | CTTGGTCTGGTGGCAGGTAT |  |  |
|  | CCAGAGACCAGGTGGGAATA | Electron transfer chain | |
|  |  | *yodB* | GTTATTCGCCCATGACTCGT |
| beta-Lactam resistance |  |  | ACGTCAAACCAGCCAAAAAC |
| *PBP2* | AAGAACGTTCCGGCAATATG |  |  |
|  | ATGCGGAACCTTCACGATAC | Cell cycle |  |
|  |  | *pleD* | GCGGCTAATGATGAAGAAGC |
|  |  |  | CCGGTTTGCTTACCCTCATA |
